# Baseline Gut Microbiome Signatures Correlate with Immunogenicity of SARS-CoV-2 mRNA Vaccines

**DOI:** 10.3390/ijms241411703

**Published:** 2023-07-20

**Authors:** Lauren Daddi, Yair Dorsett, Tingting Geng, Suresh Bokoliya, Hanshu Yuan, Penghua Wang, Wanli Xu, Yanjiao Zhou

**Affiliations:** 1 Department of Medicine, University of Connecticut Health Center, Farmington, CT 06030, USA; 2 Department of Immunology, University of Connecticut Health Center, Farmington, CT 06030, USA; 3 School of Nursing, University of Connecticut, Storrs, CT 06269, USA

**Keywords:** COVID-19, mRNA vaccines, microbiome, gut microbiome, SARS-CoV-2 mRNA vaccines, vaccine efficacy, microbial diversity, *Bilophila*, *Desulfobacterota*, immunization

## Abstract

The powerful immune responses elicited by the mRNA vaccines targeting the SARS-CoV-2 Spike protein contribute to their high efficacy. Yet, their efficacy can vary greatly between individuals. For vaccines not based on mRNA, cumulative evidence suggests that differences in the composition of the gut microbiome, which impact vaccine immunogenicity, are some of the factors that contribute to variations in efficacy. However, it is unclear if the microbiome impacts the novel mode of immunogenicity of the SARS-CoV-2 mRNA vaccines. We conducted a prospective longitudinal cohort study of individuals receiving SARS-CoV-2 mRNA vaccines where we measured levels of anti-Spike IgG and characterized microbiome composition, at pre-vaccination (baseline), and one week following the first and second immunizations. While we found that microbial diversity at all timepoints correlated with final IgG levels, only at baseline did microbial composition and predicted function correlate with vaccine immunogenicity. Specifically, the phylum Desulfobacterota and genus *Bilophila*, producers of immunostimulatory LPS, positively correlated with IgG, while *Bacteroides* was negatively correlated. KEGG predicted pathways relating to SCFA metabolism and sulfur metabolism, as well as structural components such as flagellin and capsular polysaccharides, also positively correlated with IgG levels. Consistent with these findings, depleting the microbiome with antibiotics reduced the immunogenicity of the BNT162b2 vaccine in mice. These findings suggest that gut microbiome composition impacts the immunogenicity of the SARS-CoV-2 mRNA vaccines.

## 1. Introduction

The rapid development and administration of vaccines against SARS-CoV-2 have played a critical role in the COVID-19 pandemic response. As of January 2023, over 13 billion vaccine doses against SARS-CoV-2 have been administered globally [1]. Novel mRNA vaccines have dominated COVID-19 vaccination programs due to their ability to elicit potent immune responses while allowing for rapid design and manufacturing [2]. Although the mechanisms underlying the immunogenicity of mRNA vaccines are not fully elucidated [3], its varied efficacy between individuals and reduction in efficacy among the obese, hypertensive, and elderly, resemble the patterns of conventional vaccines [4,5,6,7,8,9,10]. This suggests that the underlying variables that generally influence vaccine immunogenicity, such as primed innate immunity, may also guide the response to mRNA vaccines [11]. A full accounting of the factors that guide the immunogenicity and efficacy of mRNA vaccines will benefit at-risk individuals and populations.

The composition and function of the gut microbiome change significantly over the course of a lifetime and vary profoundly between individuals [12,13,14]. Evidence from both animal and clinical studies demonstrate that a healthy intact microbiome and production of microbial metabolites are critical for eliciting an effective immune response to vaccination [15,16,17,18,19]. Mouse studies have revealed that microbiome-derived components (e.g., LPS, DNA, and flagellin) that stimulate pattern recognition receptors, such as Toll-Like Receptors (TLRs), act as endogenous adjuvants that boost steady state innate immunity and promote the efficacy of oral and parenteral vaccines [20,21,22,23]. This appears to translate to humans, as a clinical study on the trivalent influenza vaccine found that the abundance of gut microbiota (as measured by 16S copy numbers as well as LPS and flagellin levels in stool) and microbial synthesis of specific secondary bile acids, correlated with immunogenicity in individuals with low pre-existing immunity [17]. In addition, a recent vaccinology study indicated that this may also hold true for at least 12 other vaccines, as pre-existing blood transcriptional signatures indicative of inflammatory TLR signaling, as elicited by the adjuvant, are predictive of high serum antibody responses one month after immunization [11].

Yet, experiments in mice investigating the source of adjuvant activity that mediates the high immunogenicity of the BNT162b2 mRNA vaccine found that robust antibody and CD8 T cell responses to BNT162b2 vaccination are not dependent on individual TLRs (including TLR2-5 and TLR7), nor STING, which detects cytoplasmic microbial DNA [24]. However, the functional redundancy of TLR signaling upon stimulation of different TLRs by different microbiome-derived ligands, such as flagellin and LPS, would not be revealed by these experiments. Consistent with this idea, a previous study has shown that the common downstream TLR signaling adapter Myd88 was required for optimal antibody responses to a differently formulated mRNA vaccine [25]. Additionally, a study conducted in Hong Kong on the BNT162b2 mRNA vaccine found that individuals with baseline microbiomes harboring a higher relative abundance of microbial genes associated with the expression of flagellin and fimbriae had better antibody responses, suggesting the microbiome may act as an endogenous adjuvant in this instance [26]. Further studies are required to build a consensus on how the microbiome relates to the efficacy of the mRNA vaccines across different populations.

In addition to the gut microbiome’s impact on immunity, viral infection and immunization have the potential to disrupt the composition of the microbiome. Changes in the gut microbial composition due to respiratory viral infections have been well established and include sweeping changes during COVID-19 infection [27,28,29,30,31,32,33,34,35,36]. A portion of these changes likely result from eliciting a powerful immune response, rather than viral infection itself, as respiratory viral immunization alone can induce changes in gut microbial composition [28]. To investigate whether the response to mRNA vaccination is influenced and/or altered by the gut microbiome, we conducted a pilot study including recipients of both the Moderna (mRNA-1273) and Pfizer (BNT162b2) vaccines. We analyzed the composition of the gut microbiome with 16S sequencing and measured IgG levels prior to vaccination and one week following both the first and second doses. We identified correlations between baseline microbial diversity, taxa, and predicted metabolic gene markers with IgG responses. In addition, we performed a proof-of-concept experiment in mice which demonstrates that a healthy intact microbiome boosts the immunogenicity of the Pfizer (BNT162b2) vaccine.

## 2. Results

### 2.1. Subject Demographics

**Figure 1:**
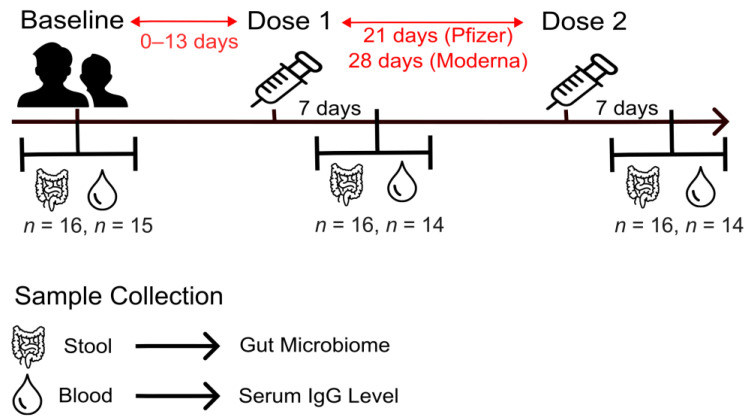
Study Design. Baseline blood and stool samples were collected from study participants up to 14 days prior to their first vaccine dose. Participants received their second vaccine dose either 21 (Pfizer) or 28 (Moderna) days after the first. Follow-up stool and blood samples were collected at 7 days post-dose 1 and dose 2.

A total of 16 healthy subjects (4 male, 12 female) participated in this study (Table 1, Figure 1). The average age at enrollment was 30.4 years, with an age range of 18–48 years. Primary series mRNA COVID-19 vaccines were received by all subjects, of whom nine (56.3%) received the vaccine manufactured by Pfizer-BioNTech (COMIRNATY) and seven (43.8%) received the vaccine manufactured by Moderna (Spikevax). Fifteen subjects chose to self-report race/ethnicity, a cohort that consisted of nine (56.3%) Caucasians and six (40%) Asians.

### 2.2. IgG Response to SARS-CoV-2 Vaccination

**Figure 2:**
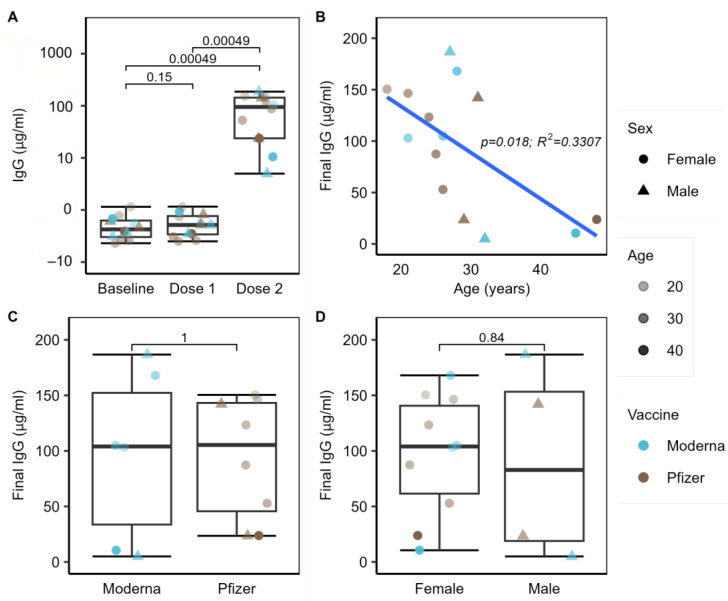
Summary of IgG responses. (**A**) Between baseline and dose 1 there was no significant increase in IgG (*p* = 0.15), however, IgG levels increased dramatically following dose 2 (*p* = 0.00049) (*n* = 15 at baseline, *n* = 14 at dose 1 and dose 2); (**B**–**D**) age significantly correlated with final IgG level. Among the 14 subjects for whom we obtained IgG levels post-dose 2, a significant negative linear relationship was observed between age and IgG response (*p* = 0.018). There were no significant IgG differences observed between the 10 male and 4 female subjects (*p* = 0.84) nor between subjects receiving the Moderna (*n* = 6) or Pfizer (*n* = 8) vaccines (*p* = 1.0). Sex is indicated by shape, vaccine manufacturer by color, and age by opacity.

Baseline IgG levels were low with a median titer of 0.46 µg/mL (IQR: 0.34, 0.73) (Figure 2A), indicating that there was little to no prior exposure to or infection with SARS-CoV-2 among the study cohort. The median titer of IgG at the second timepoint was 0.52 µg/mL (IQR: 0.35, 0.80)—an average fold-change of 0.11 µg/mL from the first timepoint—an insignificant change in IgG level (Wilcoxon *p* = 0.15). At the third timepoint, there was a significant increase in IgG (*p* = 0.00049) from both the baseline and dose 1 timepoints, with a median of 104.0 µg/mL (IQR: 31.1, 145.4). The average log-fold change between the second and third timepoints was 198.8 µg/mL. All subjects had an increase in IgG to greater than 4.0 µg/mL and were considered to have responded positively to immunization. (Figure 2A). Both participants with exposure to antibiotics in the last 3 months also responded adequately to the vaccine, with final IgG titers of 23.83 and 103.03 µg/mL.

Since it is well documented that older age groups tend to have a less robust immune response to vaccination, we fitted a linear model to our age data against IgG responses and, as expected, found a significant (*p* = 0.018, R^2^ = 0.3307) negative correlation between age and final IgG response, even among our relatively young cohort (Figure 2B). Between subjects who received the Pfizer or Moderna vaccine, there was no significant difference in their final IgG levels after the second vaccine dose (Figure 2C). There was also no statistically significant difference in IgG level by sex between the 4 male and 10 female subjects in our study from whom we obtained IgG data after the second vaccine dose (Figure 2D).

### 2.3. The Baseline Microbiome and Demographic Factors

**Figure 3:**
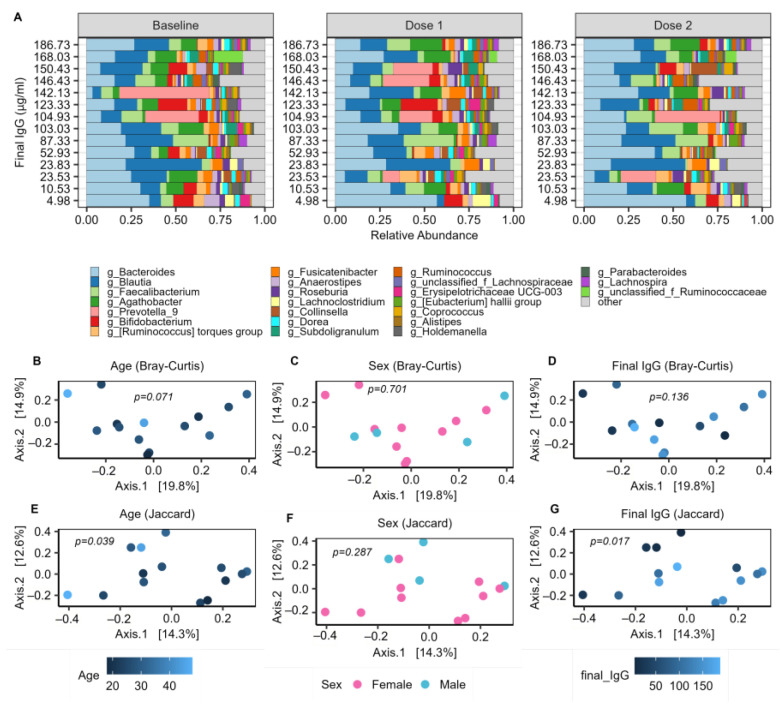
Summary of microbiome profiles. (**A**). Relative abundance of the top 25 genera across the 3 sampling timepoints; (**B**–**G**) PCoA plots of baseline microbiomes with colors to indicate subjects of varying age, sex, and final IgG level. PERMANOVA identified a significant relationship between the baseline microbiome and age (*p* = 0.039), as well as between the baseline microbiome and IgG response post-dose 2 (*p* = 0.017) with the Jaccard distance metric. All 16 subjects were included for analysis of relative abundance, age, and sex. IgG analysis included the 14 subjects from whom final IgG titers were obtained.

A total of 1,156,986 high-quality reads were obtained for an average of 17,050 reads per sample. We identified 2668 unique ASV, which consisted of 224 unique genera and 12 unique phyla. The gut microbiome in our cohort is dominated by *Bacteroides* and *Blautia*, along with several samples containing relatively high abundances of *Faecalibacterium*, *Agathobacter*, *Prevotella_9,* and *Bifidobacterium* (Figure 3A).

PERMANOVA was performed using both Bray–Curtis and Jaccard distance metrics to evaluate any demographic factors that may have significantly impacted the composition of the baseline microbiomes. There were no significant differences in baseline microbiomes between male and female subjects, or between recipients of the Moderna or Pfizer vaccines. Furthermore, microbiome beta diversity remained insignificant (*p* > 0.1) between sexes and vaccine groups at both the dose 1 and dose 2 timepoints, indicating that there was no significant difference in microbiome composition between these groups at any sampling timepoint. There was also no obvious difference in microbiome composition between the two subjects having consumed antibiotics in the last 3 months (represented by IgG levels of 23.83 and 103.03 in Figure 3A) and the rest of the study cohort. Age was significantly related to baseline microbiome composition with Jaccard distance (*p* = 0.039), and also showed an association with Bray–Curtis distance (*p* = 0.071) (Figure 3B–F). This indicates that age has a significant impact on the microbiome composition.

### 2.4. Baseline Microbial Beta-Diversity Is Associated with IgG Response

Additionally, PERMANOVA identified a significant (*p* = 0.017) correlation between the baseline microbiome and IgG using Jaccard distance (Figure 3G), indicating a significant relationship between microbiome composition and immune response to vaccination among our study population. This effect remained significant when controlling for age (*p* = 0.019), suggesting that there are variations in the microbiomes with IgG which are independent of the age variation. To then evaluate specific microbial signatures which associate with vaccination response, linear and quadratic models were constructed to examine relationships with measures of alpha diversity as well as individual taxa.

### 2.5. Microbial Alpha Diversity Is Associated with IgG Response

Linear and quadratic regression models were used to investigate relationships between IgG response and richness and Shannon diversity. Four sets of linear correlations were constructed to investigate the impact of sex and age on any identified relationships, including uncontrolled, age-controlled, sex-controlled, and age- and sex-controlled models.

Following these steps, it was found that increased baseline Shannon diversity and richness were significantly correlated with increased IgG levels. Although the correlation with richness was no longer significant when controlled for age, the relationship with Shannon diversity remained significant (*p* = 0.048). Further investigation revealed that baseline richness was significantly correlated with age (*p* = 0.03, R^2^ = 0.27), which explains why the correlation no longer held once this confounding variable was accounted for. No further linear correlations were identified at other timepoints.

**Figure 4:**
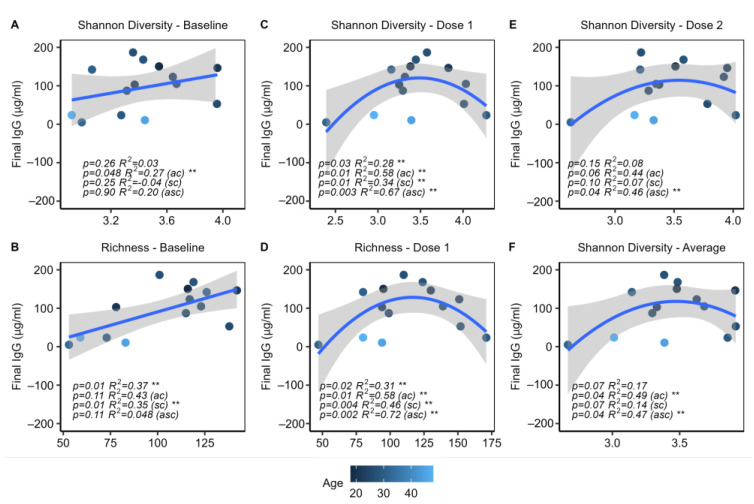
Diversity and richness are associated with IgG response. *n* = 14, including all subjects with final IgG titers. Linear and quadratic regressions with and without controlling age (ac), sex (sc), or age and sex (asc) as covariates. (**A**,**B**) Shannon diversity and richness at baseline (BL) showed positive linear correlations with the final IgG level. (**C**,**D**) Shannon diversity and richness after dose 1 showed significant quadratic relationships with the final IgG level. (**E**) Shannon diversity at dose 2 was also quadratically associated with the final IgG level. (**F**) The average Shannon diversity of each subject across all 3 timepoints showed a significant quadratic relationship with the final IgG. ** indicates *p* < 0.05.

Quadratic models were also constructed in sets of four, similar to the linear models. It was found that the average Shannon diversity of each individual across all timepoints showed a significant quadratic relationship with IgG (Figure 4A). Both richness and Shannon diversity at dose 1 showed parabolic relationships to IgG response, both with and without controlling age and/or sex (Figure 4D–E). Shannon diversity at dose 2 also showed a significant quadratic relationship with IgG response when controlled for age and sex (Figure 4F). These findings indicate that a very high or low diversity after the first or second dose appears to be associated with a poorer immune response among participants, while a high diversity prior to beginning the vaccination series is associated with an improved response.

### 2.6. Specific Microbes at Baseline Are Associated with IgG Response

**Figure 5:**
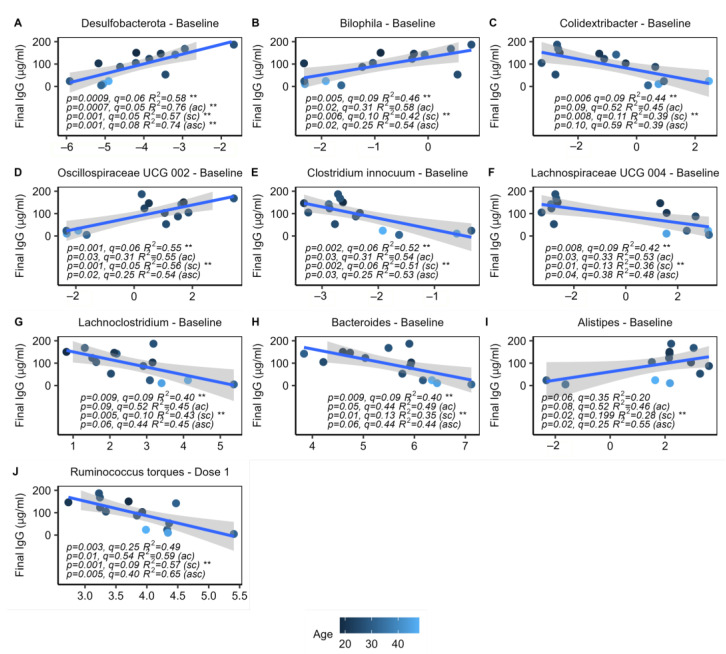
Taxa correlated with IgG response. *n* = 14, including all subjects with final IgG titers. Linear regressions with and without controlling age (ac), sex (sc), or age and sex (asc) as covariates. (**A**–**I**) Eight baseline taxa significantly correlated with the final IgG response. The genus *Bilophila* and its phylum Desulfobacterota showed significant positive correlations with final IgG (*q* = 0.09, 0.06, respectively). *Alistipes* and an unclassified Oscillospiraceae genus also positively correlated with IgG, while *Colidextribacter*, *Clostridium innocuum*, *Lachnoclostridium*, *Bacteroides*, and an unclassified Lachnospiraceae genus negatively correlated. (**J**) *Ruminococcus torques* was the only taxa at dose 1 to correlate with the final IgG response. No significant findings were found at the dose 2 timepoint. ** indicates *p* < 0.05.

Linear regression models were constructed to investigate how the baseline abundance of specific microbes may correlate with IgG response (Table S1). Models were created using CLR-transformed rarefied data and were tested both with and without controlling for age and/or sex. Nine genera and phyla at baseline significantly correlated with IgG response, including the sulfate-reducing *Bilophila* of the Desulfobacterota phylum (*q* = 0.09, 0.06), which showed positive correlations with IgG at the genus and phylum levels (Figure 5B). An unclassified genus (UCG 002) of the Oscillospiraceae family was also positively correlated with IgG, along with *Alistipes* (Figure 5D,I). Negative correlations were observed among the genera *Colidextribacter*, *Clostridium innocuum group*, *Lachnoclostridium*, and an unclassified genus (UCG 004) of the Lachnospiraceae family, as well as *Bacteroides*, a genus known to produce immunosuppressive LPS (Figure 5). The trend of decreasing baseline abundance of *Bacteroides* with improved IgG responses is visibly apparent on the bar plot of the most highly abundant genera (Figure 3A).

While there were many significant correlations with the baseline microbiome, there were no significant correlations at the dose 2 timepoint, and only 1 significant finding at the dose 1 timepoint, the genus *Ruminococcus torques group*, which showed a negative correlation with IgG (Figure 5J). Additionally, the CLR transformed abundances of each genus and phyla were averaged across all three timepoints for each individual and further tested using the aforementioned models; however, there were no significant correlations between this aggregate data and IgG. These findings suggest that the microbiome pre-vaccination is critical to eliciting an appropriate immune response.

### 2.7. Increase of Proteobacteria in Response to Vaccination Series

**Figure 6:**
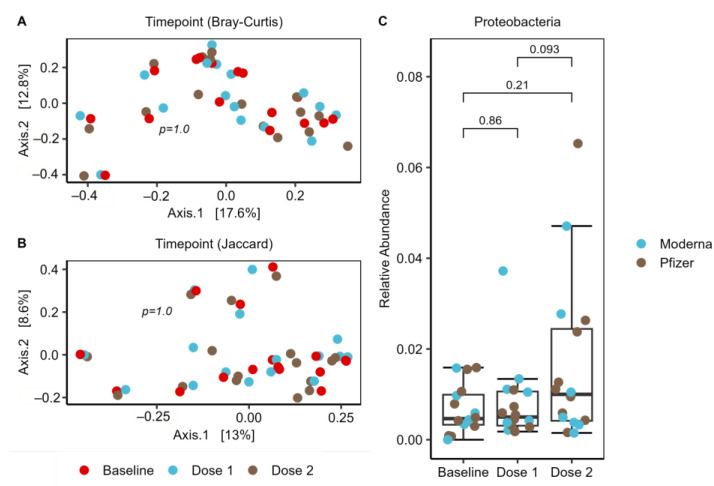
Longitudinal trends. *n* = 16, including all subjects. (**A**,**B**) PCoA plots indicate that the 3 samples from each of the 16 individual subjects tend to cluster together, indicating that each subject had a consistently distinct microbiome. PERMANOVA confirmed there was no significant change in the microbiome over the vaccination course (*p* = 1.0); (**C**) upon using DESeq2 to evaluate individual taxa, a significant increase in the phylum Proteobacteria after dose 2 was observed (log2fold change = 1.24), which was confirmed as a trend with a Wilcox test (*p* = 0.093).

To investigate changes in the microbiome over the course of the vaccination series, PERMANOVA was performed using both Bray–Curtis and Jaccard distance metrics, with no significant differences observed by timepoint (*p* = 1.0). This was further visualized with PCoA plots using these distance metrics. While there was no clustering by timepoint, the three samples from each subject tend to cluster together, indicating that each subject had a unique microbiome that remained relatively consistent over the vaccination course, which was an expected finding (Figure 6A,B). To investigate any changes in differential abundance which may have occurred during vaccination, DESeq2 was performed at the ASV, genus, and phylum levels. A significant increase in Proteobacteria, a phylum known to produce immunostimulatory LPS [37], was identified in the second vaccine dose (log2 fold change = 1.24, adjusted *p* = 0.04) (Figure 6C). The DESeq2 finding was followed up with a paired Wilcoxon rank-sum test (*p* = 0.093) and visualized on a boxplot, which provided further support for a trend of heightened Proteobacteria after vaccination. No other significant findings were found, indicating that the relative abundance of most microbes, except Proteobacteria, remained relatively consistent over the vaccination course.

### 2.8. Predicted Metabolic Functions Correlate with IgG Response

KEGG orthologs, Enzyme Classification numbers (ECs), and metabolic pathways predicted from PICRUSt2 were further analyzed at each timepoint to evaluate any correlations with IgG. Linear models were constructed, and it was found that the predicted metabolic functions at baseline tended to be much more greatly correlated with IgG response than at the other timepoints, as only correlations from this timepoint showed both significant *p*-values and FDR adjusted *q*-values, indicating that once again, the baseline microbiome appears to be most critical to eliciting an appropriate immune response (Table S2).

**Figure 7:**
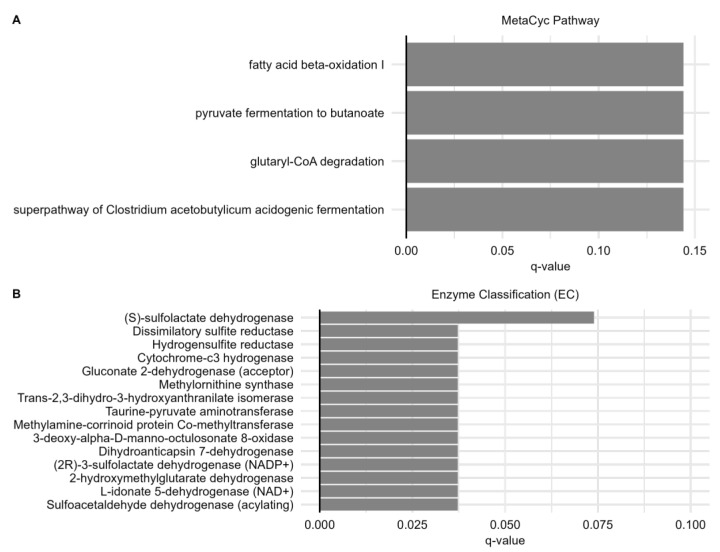
Predicted metabolic functions correlate with the final IgG response. (**A**) Many baseline KEGG predicted metabolic function markers, including four metabolic pathways. (**B**) Fifteen enzyme classifications (ECs) correlated with final IgG levels. No significant findings were found at the dose 1 or dose 2 timepoints.

There were 144 significant (*q* < 0.2) baseline metabolic signatures positively correlated with IgG, including 4 metabolic pathways, 15 ECs (Figure 7), and 124 KOs (Figure S1) (Table S2). The most significant finding was a positive correlation between the KO sulfoacetaldehyde dehydrogenase and IgG response, which is consistent with the sulfate-reducing microbes Desulfobacterota and *Bilophila* being most strongly associated with IgG. At the EC level, a sulfoacetaldehyde dehydrogenase, two sulfolactate dehydrogenases, hydrogensulfite reductase, and dissimilatory sulfite reductase were positively associated with IgG. Additionally, many markers associated with the type-3 secretion system, flagellin, and capsular polysaccharides were positively correlated with IgG, particularly at the KO level, suggesting a possible immunostimulatory mechanism via these microbial signatures. Furthermore, several pathways related to short-chain fatty acid metabolism at baseline correlated with IgG response.

### 2.9. The Intact Gut Microbiome Is Important for Optimal Vaccination Response in Mice

**Figure 8:**
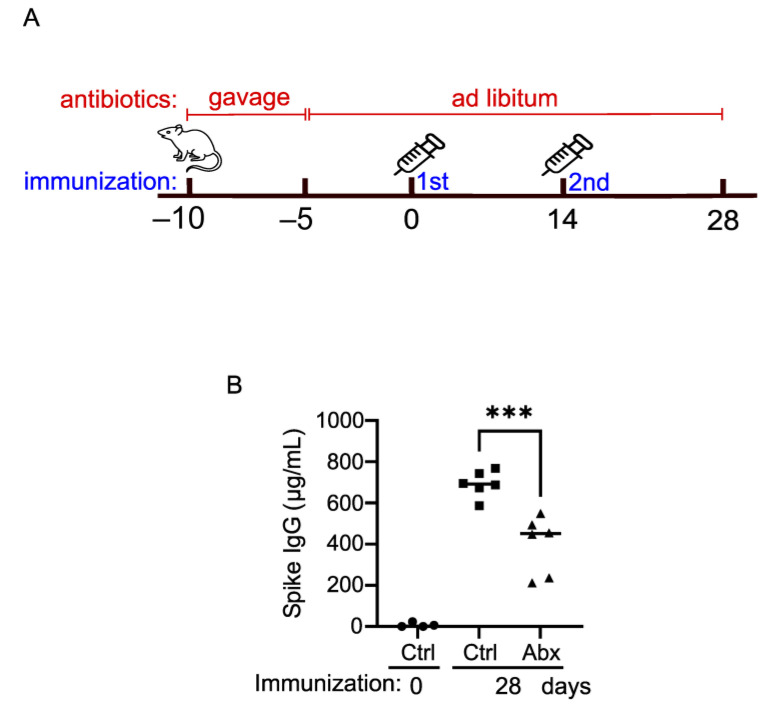
The gut microbiome contributes to optimal vaccination efficacy. (**A**) Timeline of antibiotic treatment and immunization; (**B**) concentrations of serum anti-SARS-CoV-2 spike IgG. Each dot = one animal, with squares indicating control mice and triangles indicating antibiotic-treated mice. *p* < 0.001 (one-way ANOVA). Ctrl: control mice treated with water instead of antibiotics in the same way. *** indicates *p* < 0.001.

To corroborate the clinical findings, we next examined the impact of the gut microbiome on COVID-19 vaccine efficacy with a mouse model. We assessed the Pfizer COVID-19 mRNA vaccine-induced anti-SARS-CoV-2 Spike IgG production when the gut microbiome was depleted with a cocktail of antibiotics (Abx). Compared to those in the control mice (Ctrl), the serum Spike IgG concentrations in the Abx mice were reduced by ~30% (Figure 8), suggesting that the gut microbiome is important for optimal vaccination efficacy.

## 3. Discussion

Our study supports the hypothesis that the gut microbiome boosts the immunogenicity of mRNA vaccines as it does for conventional vaccines. We identified a positive relationship between both microbial diversity and several microbial taxa at baseline with final levels of IgG. These included positive correlations with Desulfobacterota and *Bilophila*, and a negative correlation with *Bacteroides*. In addition, we noted positive associations between several predicted metabolic gene markers at baseline and IgG response, including pathways associated with short-chain fatty acid (SCFA) synthesis and markers of sulfur metabolism. Consistent with a role for the microbiome as an endogenous adjuvant, predictive gene ontology analysis also identified strong positive correlations between IgG levels and several bacterial structural components such as flagellin and capsular polysaccharides. As a follow-up to our human study, we performed a proof of principle experiment in mice, which demonstrated that an intact microbiome may be important for optimal mRNA vaccination immunogenicity.

It is well documented that the efficacy of conventional vaccines and mRNA vaccines can vary with chronological age, generally decreasing over the course of a lifetime [4,5,6,7,8,38,39]. Higher alpha diversity is generally associated with a healthy gut microbiome compared to disease conditions. However, recent findings suggest that alpha diversity of the gut microbiome increases with age, and that exceedingly high diversity is evidence of reduced colonization resistance due to poor health [40,41,42]. Despite this, we found that baseline levels of microbial diversity and richness were linearly correlated with the final IgG response, even after controlling for age in the case of diversity. Yet, at dose 1, there was an age-independent parabolic relationship between IgG levels, and microbial diversity and richness. Similar, yet weaker, diversity findings were observed at the dose 2 timepoint and with aggregate data. This relationship suggests that there may exist an optimal microbial diversity and richness for promoting effective IgG responses, which likely reflects the overall health of the individual [43].

In moving forward with our analysis, we continued to note which of the many correlations we identified were accompanied by a relationship with age. While the age variance complicates the interpretation of our findings, it does not dismiss the potential for a true relationship between our identified microbial signatures and vaccine response, as the effect of age on vaccination response may potentially be mediated by age-related changes in the microbiome [17]. To deconstruct these relationships, we further evaluated the role of the microbiome as an independent factor influencing IgG response by controlling for age in our analysis. Although some studies have observed a sex difference in COVID-19 vaccine response [44], we did not observe any significant differences in microbiome composition or IgG response between males and females or between recipients of the Moderna and Pfizer vaccines, potentially due to a small sample size.

While we found correlations between microbial diversity/richness and immunogenicity across all timepoints, correlations with individual taxa existed primarily at baseline. Specifically, we found that levels of the genus *Bilophila* and its phylum Desulfobacterota, taxa associated with the Western diet, and consumption of animal products [37,45] correlated with IgG response for both mRNA vaccines. Although a recent study on the inactivated COVID-19 vaccine did not identify a correlation between *Bilophila* and IgG levels, it did find that this genus had the greatest impact on the activation of numerous immune cell subsets [19]. Among all the taxa that correlated with IgG in our study, only Desulfobacterota remained significant after controlling for age.

Desulfobacterota is a newly designated phylum containing members reclassified from the Thermodesulfobacteria phylum and Deltaproteobacteria class of the phylum Proteobacteria [37]. *Bilophila*, a prior member of Deltaproteobacteria, synthesizes immunostimulatory endotoxin (LPS), a common property among Proteobacteria [37]. Consistent with the prominent role of bacterial LPS in modulating vaccine immunogenicity, we found that baseline levels of *Bacteroides*, which contains immunosuppressive LPS [46], negatively correlated with IgG. Epidemiological data have linked the ratio of Enterobacteriaceae (a family within the phylum Proteobacteria) to *Bacteroides* with vaccine immunogenicity, and this is believed to be representative of their respective LPS properties [15]. Experimental evidence in support of the LPS theory is found in proof of principle experiments which successfully boosted responses to an oral rotavirus vaccine in humans by increasing the relative abundance of Proteobacteria with narrow-spectrum antibiotics [16].

A recent study found that baseline pro-inflammatory transcriptional signatures associated with innate immunity are a general predictor of vaccine immunogenicity [11]. Therefore, the proinflammatory properties of *Bilophila* may act to prime immune responses to many types of vaccines. In support of this idea, a very recently published study identified a strong positive correlation between baseline levels of *Bilophila*, which is associated with IBD, and final levels of anti-spike IgG in immunosuppressed IBD patients vaccinated with BNT162b2 or ChAdOx1 [47]. However, several studies have found specific commensal microbiota that induces antibodies that cross-react with the RBD domain of the spike protein, and therefore prime immune responses to both SARS-CoV-2 infection as well as SARS-CoV-2 vaccines [48,49,50]. Intriguingly, a relatively recent work in bioRxiv indicates that *Bilophila* may in fact support immune responses specific to SARS-CoV-2, as pre-existing IgA and IgG anti-RBD antibodies in healthy individuals bound only to *Bilophila* and Parabacteroides [51]. Such a role for *Bilophila* is consistent with the finding that the relative abundance of *Bilophila* is inversely correlated with COVID-19 disease severity [52].

However, caution against using *Bilophila* for therapeutic purposes in COVID-19 is warranted as its relative abundance is increased in COVID-19 patients treated with antibiotics [53], and antibiotic treatments may result in mortality via translocation of commensal microbiota and bacteremia [27]. In agreement, a major risk factor for mortality of COVID-19 patients admitted to the ICU is an increased abundance of *Bilophila* within 60 days of being admitted to the ICU [54]. This detrimental aspect of *Bilophila* is likely reflective of its proinflammatory properties [55,56], which may promote the cytokine storms that underly severe COVID-19 [57,58].

In addition to LPS, *Bilophila* can produce metabolites that interact with the immune system. Our 16S rRNA gene sequence prediction identified many positive correlations between gene ontology terms and final IgG response, including positive correlations with markers of taurine and sulfur metabolism, which are characteristic of *Bilophila*. *Bilophila* preferentially grows in bile-rich environments by utilizing the sulfur in taurine-conjugated bile acids, which are preferentially synthesized (over glycin-conjugated bile acids) upon consumption of a typical Western diet [37]. That Desulfobacterota was the only correlation that remained significant after controlling for age and that Oscillospiraceae, a family of microbes that also synthesize bile acids, correlate with IgG emphasizes the potential importance of bile acid levels and vaccine efficacy. This is highly relevant given the putative role of microbial-produced bile acids in modulating the efficacy of the flu vaccine [17].

Our gene ontology analysis also revealed positive correlations between gene markers for incorporation of flagellin and piliae, as well as capsular polysaccharides, with final levels of IgG. This is consistent with a recent study on the Pfizer vaccine in Hong Kong, which identified a positive correlation between the enrichment of cell motility genes and IgG levels approximately one month after the second dose [26]. This finding is also supported by experimental data from mice that revealed that commensal flagellin acts as an endogenous adjuvant for the trivalent influenza vaccine [20]. The potential link to capsular polysaccharides is interesting considering they have been shown to modulate responses to other vaccines and can play diverse immunomodulatory roles through interaction with Toll-like receptors (TLRs) [59]. At the pathway level, we identified positive correlations between short-chain fatty acids (SCFAs) synthesis and IgG response. This is consistent with the recent manuscript on the inactivated COVID-19 vaccine [19], which identified high levels of SCFA pathways and SCFA levels at baseline as the strongest microbial feature predictive of anti-Ace2 antibody levels following immunization.

Throughout our study, most of the correlations we identified were between baseline microbial signatures (richness, diversity, taxonomic abundance, metabolic function) and final levels of IgG. This is consistent with a study of the inactivated COVID-19 vaccine, which showed that correlations between microbiota and IgG occurred primarily at baseline [19]. Comparable results were found in a clinical study of the parenteral trivalent inactivated influenza vaccine, which revealed that the role of the microbiome in promoting vaccine efficacy was restricted to individuals with low pre-existing immunity, as opposed to individuals with high immunity due to prior antigen exposure [17]. Taken together, the findings from these studies suggest that microbiome composition and function sensitize primary immune responses, but do not significantly impact secondary responses as memory cells are more sensitive to activation by cognate antigen.

Our study expands the work on the BNT162b2 vaccine previously performed in Hong Kong [26] by including the Moderna vaccine and by analyzing a new geographically distinct cohort. Furthermore, our study adhered to a strict three-point timeline that investigated the impact of the microbiome on secondary immunization and analyzed IgG levels at approximately seven days post-immunization. This addressed a shortcoming of the Hong Kong study, which only analyzed the microbial composition and antibody titers at baseline and one month following the second dose, which may have allowed undefined variables to weaken any existing correlations.

The data from our human study suggest that differential gut microbiome composition and function can influence IgG response to mRNA vaccination. We confirmed this observation by conducting a proof-of-principle experiment in mice, where we observed that disruption of the microbiome by antibiotic administration resulted in a 30% reduction of IgG levels as compared to controls. This suggests that mRNA vaccines may depend on sensing the host microbiome in a manner similar to conventional unadjuvanted vaccines. We emphasize that larger, more rigorous studies are required to control for any off-target effect of antibiotics on immune responses [60].

The primary limitation of our study was the recruitment of a small sample size. This limited our ability to identify smaller effects or to generalize our findings to larger, more diverse patient populations, as our cohort lacked representation of older age groups and diverse racial and geographic backgrounds. Despite this limitation, our study was representative of numerous larger studies, including the Hong Kong study (Pfizer *n* = 101), in terms of the distribution of IgG responses between individuals with respect to age. Furthermore, due to the nature of 16S rRNA sequencing, our assessment of metabolic function markers relied on predictive metagenomes as opposed to direct genomic data or metabolomics. Lastly, the mice experiment served as a proof-of-concept study. Only young adult, female mice were included. This may prevent the generalization of findings to males and elder mice. However, due to the strong impact of antibiotics on the gut microbiome, we expect a decrease in IgG response, but the degree of decrease may vary by sex and age [44,61].

The findings from our cohort study and mouse experiment indicate that there is a role for the microbiome in eliciting a proper response to mRNA vaccination. We emphasize the importance of continued research on large diverse study populations, coupled with additional controlled experiments on animal models, to elucidate the molecular interactions at play. This research remains critical for understanding and optimizing the efficacy of mRNA vaccines, our most effective tool currently available to combat COVID-19.

## 4. Materials and Methods

### 4.1. Study Cohort and Sample Collection

A prospective cohort study was conducted to recruit COVID-19 vaccine-naive volunteers from the northeast U.S. between March 2021 and October 2021. Adult men and women who were scheduled to receive the COVID-19 mRNA vaccine, English speaking, and were able to provide informed consent were invited to participate. We excluded individuals who had received any COVID-19 vaccine before; received blood product within three months of enrolling in the study; had a recent COVID-19 infection (within the past six months); were receiving steroids by mouth or systematically; were pregnant; or had chronic neurological conditions or autoimmune disease. None of the participants enrolled in our study were currently taking metformin or proton pump inhibitor medications.

Upon consent, participants were invited to the biobehavioral lab for three investigational visits: within 14 days (range 0–13 days) prior to the first dose of the COVID-19 mRNA vaccine and 7 ± 3 days after the first and the second doses of the COVID-19 mRNA vaccine. The time in between doses was 21 days for Pfizer (BNT162b2) recipients and 28 days for Moderna (mRNA-1273) recipients. Survey questionnaires were completed at the beginning of the study to provide information on demographic characteristics, health factors, and medical/medication history. Venous blood samples (10 mL) were collected in EDTA tubes during each visit and were immediately centrifuged to obtain plasma. Stools were collected using OMNIgene^®^GUT (OMR-200) home collection kits (DNA Genotek Inc., Ottawa, ON, Canada) within 48 h of each visit and mailed back to the lab, where the samples were immediately aliquoted and stored at −80 °C.

### 4.2. Quantification of Serum IgG by Enzyme-Linked Immunosorbent Assay (ELISA)

The human anti-SARS-CoV-2 Spike IgG titers were measured with a commercial ELISA kit (Biolegend, San Diego, CA, USA, Cat # 447807). The human sera were diluted by 1000-fold (timepoint 1 and 2) or 10,000-fold (timepoint 3). Thereafter, 50 µL of each diluted sample and 50 µL of Assay Buffer B were added to a 96-well plate and incubated at room temperature for 2 h. The wells were washed four times with the wash buffer. 100 µL of SARS-CoV-2 Spike S1 Human IgG Detection Antibody solution was added to each well and incubated at room temperature for 1 h. After four washes, 100 µL of Avidin-HRP solution was added to each well and incubated at room temperature for 30 min. After stringency washes, 100 µL of substrate 3,3′,5,5′-tetramethylbenzidine (TMB) was added to each well and incubated at room temperature for 5–30 min for color development and terminated with 100 µL of Stop Solution. The absorption at wavelength 450 nm (A450 nm) was read on a Cytation 1 plate reader (BioTek, Winooski, VT, USA).

For mouse studies, an ELISA kit from Acro Biosystems, Newark, DE, USA, Cat # RAS-T018, was applied to the titration of mouse serum IgG. Briefly, 100 µL of each diluted serum (10,000-fold) or standard was added to a 96-well plate and incubated at room temperature for 1 h. The wells were washed four times with the wash buffer. Next, 100 µL of diluted horseradish peroxidase (HRP)-conjugated goat anti-mouse IgG was added to each well and incubated at room temperature for 1 hr. The rest of the procedures were the same as above.

### 4.3. Mouse Vaccination

Approximately seven-week-old female C57BL/6 mice (*n* = 6) were orally gavaged with 10 mg of an antibiotic cocktail containing vancomycin, neomycin, metronidazole, and ampicillin once a day for 5 days, and then fed ad libitum with antibiotic water which contained 1 mg/mL each of neomycin, metronidazole, and ampicillin and 0.5 mg/mL of vancomycin throughout the experimentation. The control group (*n* = 6) was sex- and age-matched mice treated with water in the same way. Five days after the last dose of oral gavage, pre-immune sera were collected from 4 mice as a baseline measurement, and 4 µg of COVID-19 mRNA vaccine (Pfizer) was injected intramuscularly into one high leg. The second dose was given 14 days after the first dose. The immune sera were collected 28 days after the first dose for subsequent immunological analyses.

### 4.4. DNA Extraction, 16S rRNA Gene Sequencing and Data Processing

DNA was isolated from human stool samples using the ZymoBIOMICS^®^ DNA Miniprep Kit. Isolated DNA samples were quantified using a Qubit 2.0 Fluorometer and normalized to 2 ng/µL. The bacterial 16S rRNA genes were amplified via PCR using a 515F forward primer for all samples and a unique bar-coded 806R reverse primer for each individual sample. Amplified PCR products were visualized under UV light on an agarose gel using the SYBR Safe DNA stain and then purified using the Zymo Select-a-Size MagBeads. Purified 16S libraries were quantified using the Qubit, and then equal masses of amplicons from each sample were pooled. Two clean-up steps were performed on the pooled libraries using the Zymo Select-a-Size MagBeads kit. Purified libraries were then visualized on an agarose gel and quantified via qPCR using the Illumina Library Quantification Kit (ROX Low qPCR Mix Cat #E7630). Sequencing of the 16S rRNA gene was performed on an Illumina MiSeq using the MiSeq Reagent Kit v2 (2 × 250 reads, 500 cycles, Cat #MS-102-2003).

Raw sequencing reads were demultiplexed using Illumina bcl2fastq2 Conversion Software (v2.20) and RTA (v.1.18.54.4). Reads in each sample were processed using the DADA2 (v1.22.0) pipeline [62] for taxonomy assignment to amplicon sequence variants (ASVs) based on the Silva reference database (version 138.1). Our sequencing run included DNA extraction controls, PCR negative controls, and positive controls. All negative controls obtained less than 900 reads and positive controls had over 20,000 reads.

### 4.5. Metabolic Pathway Analysis Based on 16S Data

The PICRUSt2 (Phylogenetic Investigation of Communities by Reconstruction of Unobserved States) software (v2.5.1) [63] was used to analyze the functional potential of the microbiome data. ASVs with less than 10% prevalence were excluded from the data analysis. Linear models were constructed to evaluate correlations between IgG and the abundance of KEGG orthologs, Enzyme Classification numbers, and inferred MetaCyc pathways.

### 4.6. Statistics

Analyses were performed with 48 samples which covered 16 subjects across 3 timepoints. Statistical analysis, calculation of diversity metrics, bar plots, and construction of Figure 2, Figure 3, Figure 4, Figure 5 and Figure 6 were performed with R version 4.1.0 in the Rstudio interface version 1.4.1106 with the following packages: compositions (v.2.0–2), DESeq2 (v.1.32.0), ggh4x (v.0.2.0), ggplot2 (v.3.3.6), ggpubr (v.0.4.0), knitr (v.1.33), microbiome (v.1.14.0), microViz (v.0.9.0), phyloseq (v.1.36.0), stats (v.4.1.0), tibble (v.3.1.2), and vegan (v.2.5–7). Figure 1 was constructed using Inkscape (v1.2) software with open-source vector images from the UXWing site.

IgG titers over 3 timepoints were compared by paired Wilcoxon rank-sum tests. To determine the differential distribution of IgG levels among categorical sample variables (i.e., sex and vaccine manufacturer), Wilcoxon rank-sum tests were performed. To evaluate the relationship between IgG and age, a linear regression was performed.

To visualize the microbiome composition, samples were rarefied to 10,000 reads and converted to relative abundance. Stacked bar plots were constructed from the top 24 genera using the compositional bar-plot function in the microViz package [64]. All additional taxa were grouped in an additional category as “other”.

Alpha diversity was determined using the Richness and Shannon diversity metrics, computed using the alpha function in the microbiome package. Linear and quadratic models were constructed in R to evaluate correlations between alpha diversity and IgG response and were controlled with age and sex as covariates. Significant trends were visualized with scatterplots which included the corresponding linear or quadratic regression model and relevant correlation statistics.

Beta diversity was computed on rarefied ASV count data using the Bray-Curtis and Jaccard distance metrics using the distance function in the phyloseq package [65]. PERMANOVA was performed using the Adonis function in the vegan package to evaluate statistically significant differences in beta diversity across sample variables. Principal Coordinates Analysis (PcoA) was performed to visualize beta-diversity differences using the ordination functions in phyloseq.

Differential abundance of taxa was evaluated at the ASV, genus, and phylum levels using the DESeq2 package [66], with differences represented as log2 fold change. Paired two-sample Wilcoxon tests were performed to further confirm statistical significance.

To evaluate correlations between the abundance of specific taxa (>20% prevalence) in baseline microbiomes and IgG response, a center-log ratio (CLR) transformation of the relative abundance of the microbiome was performed. Linear models were constructed on the resultant data set and were controlled with age and sex as covariates. The resulting *p*-values were adjusted for multiple comparisons with the Benjamini–Hochberg correction, and then scatter plots were constructed for correlations with FDR-adjusted *p*-values (*q*-values) less than 0.20.

## Figures and Tables

**Table 1:** Characteristics of the study population.

	Subjects (*n* = 16)
Sex (M:F)	4:12
Age (median [IQR])	27.5 [24.8–34.3]
Vaccine manufacturer	
Pfizer	9
Moderna	7
Race	
White/Caucasian	9
Asian	6
Unspecified	1
Antibiotic intake (past 3 months)	2
Probiotic intake (past 2 weeks and/or currently)	2
Blood sample collected for IgG titer post-dose 2	14
Matching stool samples collected at baseline, post-dose 1, and post-dose 2	16

## Data Availability

The data presented in this study are available on request from the corresponding author.

## Supplementary Materials

The supporting information can be downloaded at: https://www.mdpi.com/article/10.3390/ijms241411703/s1.
