# Supplementary material for: Baseline Gut Microbiome Signatures Correlate with Immunogenicity of SARS-CoV-2 mRNA Vaccines
Source: Int J Mol Sci. 2023 Jul 20;24(14):11703. doi: 10.3390/ijms241411703 (PMC10380288; doi:10.3390/ijms241411703)
Supplement: Supplementary file 1 [file ijms-24-11703-s001.zip › supplemental.legends.docx]

**Figure S1.** Predicted KOs which correlate with final IgG response. 124 positive correlations with IgG were identified for baseline predicted KOs.

**Table S1.** Taxa correlations with IgG. Linear models controlled for age, sex, age and sex, and uncontrolled were constructed to evaluate the genera and phyla which correlate with final IgG levels. FDR adjusted and un-adjusted *p* values are shown along with R squared values.

**Table S2**. PICRUSt2 predicted metabolic function correlations with IgG. Linear models were constructed to evaluate the KOs, ECs, and metacyc pathways which correlate with final IgG levels. FDR adjusted and un-adjusted *p* values are shown along with R squared values.
