# Supplementary figures and images for: Baseline Gut Microbiome Signatures Correlate with Immunogenicity of SARS-CoV-2 mRNA Vaccines
Source: Int J Mol Sci. 2023 Jul 20;24(14):11703. doi: 10.3390/ijms241411703 (PMC10380288; doi:10.3390/ijms241411703)

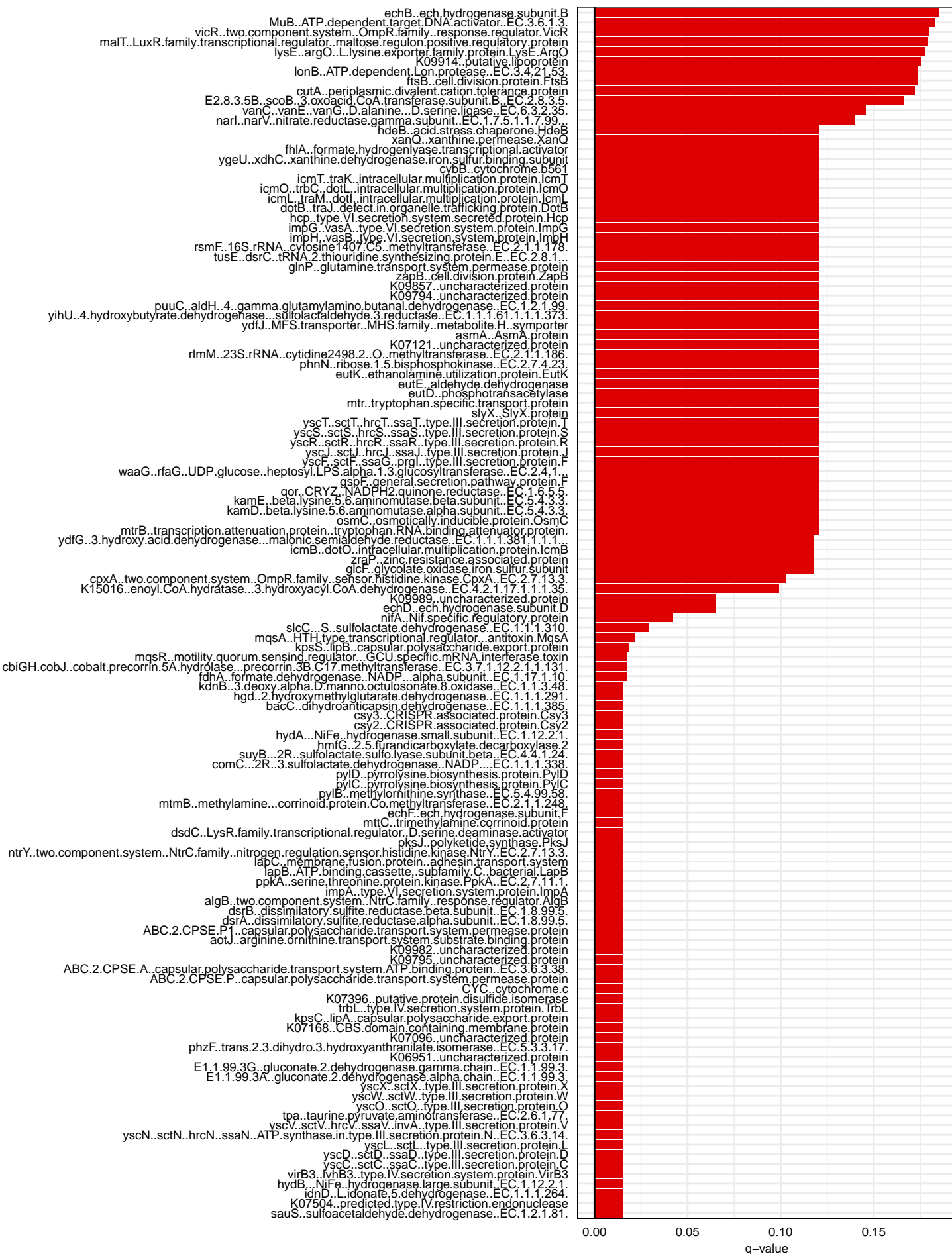

Supplement: Supplementary file 1 [file ijms-24-11703-s001.zip › figureS1_1_25_23.pdf]
